# Case of Tuberculous Ulceration of the Intestine

**Published:** 1878-04

**Authors:** W. T. Belfield


### VIII.

### CASE OF TUBERCULOUS ULCERATION OF THE INTESTINE.

By Dr. Belfield.

M. B——, a Hollander, 54 years old, was admitted to the hospital March 9, 1878. The lack of a common language prevented the extraction of his previous history. He was quite emaciated and feeble, his appetite and sleep were poor; he was troubled with persistent diarrhœa. We found consolidation of the upper lobe of the right lung, with a small cavity at the apex; also some infiltration at the left apex. There was considerable tenderness and some pain over the abdomen, especially in the right iliac region. He rapidly declined, and died March 19.

An examination showed cheesy consolidation of the upper lobe of the right lung; a small cavity at its apex; a considerable quantity of turbid serum in the peritoneal sac; fatty degeneration of the liver; caseous enlargement of the mesenteric lymph-glands and cheesy degeneration and ulceration of the solitary and agminated glands.

The latter lesions were the objects of especial interest. The enlarged and ulcerated glands were found at intervals of one to four inches along the course of the ileum, most numerous near the ileo-caecal valve. In many instances these glands had suffered not only enlargement but also softening and disintegration, and there had resulted the oval, transversely-placed ulcers which are so often (and improperly) styled tuberculous. These ulcers varied in size ; the largest, which had evidently resulted from the fusion of several smaller ulcers, extended around the greater part of the intestinal mucous circumference.

These ulcers, while primarily scrofulous, were secondarily tuberculous ; in other words the absorption of the products of the glandular inflammation had caused inflammatory action at various points along the lymphatic vessels leading from the ulcers to the mesenteric glands. The results of this inflammatory action appeared as gray, miliary nodules (tubercles) which thickly studded the subperitoneal coat of the intestine at the sites of the ulcers, and thence staked out the course of the lymphatic vessel around the intestine and across the mesentery to the mesenteric glands.

This case presents several points of interest : 1—The concurrence of pulmonary and intestinal consumption ; in other words, cheesy degeneration of inflammatory products in both air-vesicles and lymph-glands. 2—The association of miliary tubercle with the intestinal but not with the pulmonary consumption. 3—The admirable demonstration of the fact that tubercle "belongs," as Buhl says "to the lymphatic system ;" and furthermore that it is developed in lymphatic channels through which there pass irritant substances (proved by the inflamed condition of the mesenteric glands in which the lymphatic channels terminated.)

---

I take pleasure in announcing that Dr. W. T. Belfield will hereafter assist me in editing the Pathological Transactions of the Chicago Medical Society, and in acknowledging his assistance in reporting matter for the present issue. The present number of the Transactions is almost entirely made up of cases which have been used to illustrate my weekly lectures in the Necropsy Theatre of Cook County Hospital, and have been reported for the Society by Dr. Belfield.

I. N. Danforth.

